# Supplementary material for: Rfam 15: RNA families database in 2025
Source: Nucleic Acids Res. 2024 Nov 11;53(D1):D258–67. doi: 10.1093/nar/gkae1023 (PMC11701678; doi:10.1093/nar/gkae1023)
Supplement: gkae1023_Supplemental_File [file gkae1023_supplemental_file.docx]

| Rfam Accession | Rfam ID | miRBase Family Accession |
| --- | --- | --- |
| RF00027 | let-7 | MIPF0000002 |
| RF00047 | mir-2 | MIPF0000049 |
| RF00051 | mir-17 | MIPF0000001 |
| RF00052 | lin-4 | MIPF0000303 |
| RF00053 | mir-7 | MIPF0000022 |
| RF00073 | mir-156 | MIPF0000008 |
| RF00074 | mir-29 | MIPF0000009 |
| RF00075 | mir-166 | MIPF0000004 |
| RF00076 | mir-181 | MIPF0000007 |
| RF00103 | mir-1 | MIPF0000038 |
| RF00104 | mir-10 | MIPF0000033 |
| RF00104 | mir-10 | MIPF0000025 |
| RF00104 | mir-10 | MIPF0000268 |
| RF00104 | mir-10 | MIPF0000271 |
| RF00129 | mir-103 | MIPF0000024 |
| RF00130 | mir-192 | MIPF0000063 |
| RF00131 | mir-30 | MIPF0000005 |
| RF00143 | mir-6 | MIPF0000119 |
| RF00144 | mir-199 | MIPF0000040 |
| RF00237 | mir-9 | MIPF0000014 |
| RF00239 | mir-124 | MIPF0000021 |
| RF00241 | mir-8 | MIPF0000019 |
| RF00244 | mir-26 | MIPF0000043 |
| RF00245 | mir-19 | MIPF0000011 |
| RF00246 | mir-135 | MIPF0000028 |
| RF00247 | mir-160 | MIPF0000032 |
| RF00251 | mir-219 | MIPF0000044 |
| RF00253 | mir-101 | MIPF0000046 |
| RF00254 | mir-16 | MIPF0000006 |
| RF00255 | mir-218 | MIPF0000026 |
| RF00256 | mir-196 | MIPF0000031 |
| RF00257 | mir-194 | MIPF0000055 |
| RF00258 | mir-130 | MIPF0000034 |
| RF00363 | mir-BART1 | MIPF0000325 |
| RF00365 | mir-BHRF1-1 | MIPF0000331 |
| RF00366 | mir-BHRF1-2 | MIPF0000332 |
| RF00446 | mir-133 | MIPF0000029 |
| RF00455 | mir-15 | MIPF0000006 |
| RF00637 | mir-276 | MIPF0000124 |
| RF00638 | MIR159 | MIPF0000010 |
| RF00639 | mir-515 | MIPF0000020 |
| RF00640 | MIR167 | MIPF0000023 |
| RF00641 | mir-154 | MIPF0000018 |
| RF00642 | mir-23 | MIPF0000027 |
| RF00643 | MIR171_1 | MIPF0000030 |
| RF00644 | mir-27 | MIPF0000036 |
| RF00645 | MIR169_2 | MIPF0000037 |
| RF00646 | mir-204 | MIPF0000042 |
| RF00647 | MIR164 | MIPF0001794 |
| RF00648 | MIR396 | MIPF0000047 |
| RF00649 | mir-128 | MIPF0000048 |
| RF00650 | mir-153 | MIPF0000050 |
| RF00651 | mir-221 | MIPF0000051 |
| RF00652 | MIR478 | MIPF0000052 |
| RF00653 | mir-22 | MIPF0000053 |
| RF00654 | mir-216 | MIPF0000054 |
| RF00655 | mir-28 | MIPF0000057 |
| RF00656 | mir-205 | MIPF0000058 |
| RF00657 | mir-184 | MIPF0000059 |
| RF00658 | mir-21 | MIPF0000060 |
| RF00659 | mir-365 | MIPF0000061 |
| RF00660 | mir-214 | MIPF0000062 |
| RF00661 | mir-31 | MIPF0000064 |
| RF00662 | mir-132 | MIPF0000065 |
| RF00663 | mir-183 | MIPF0000066 |
| RF00664 | mir-223 | MIPF0000067 |
| RF00665 | mir-290 | MIPF0000068 |
| RF00666 | mir-32 | MIPF0000069 |
| RF00667 | mir-33 | MIPF0000070 |
| RF00668 | mir-302 | MIPF0000071 |
| RF00669 | mir-96 | MIPF0000072 |
| RF00670 | mir-105 | MIPF0000074 |
| RF00671 | mir-138 | MIPF0000075 |
| RF00672 | mir-190 | MIPF0000076 |
| RF00673 | mir-217 | MIPF0000077 |
| RF00674 | mir-187 | MIPF0000078 |
| RF00675 | mir-145 | MIPF0000079 |
| RF00676 | mir-127 | MIPF0000080 |
| RF00677 | MIR168 | MIPF0000081 |
| RF00678 | mir-140 | MIPF0000085 |
| RF00679 | mir-210 | MIPF0000086 |
| RF00680 | mir-224 | MIPF0000088 |
| RF00681 | mir-198 | MIPF0000090 |
| RF00682 | mir-144 | MIPF0000093 |
| RF00683 | mir-143 | MIPF0000094 |
| RF00684 | mir-122 | MIPF0000095 |
| RF00685 | mir-36 | MIPF0000096 |
| RF00686 | mir-338 | MIPF0000097 |
| RF00687 | mir-136 | MIPF0000099 |
| RF00688 | MIR394 | MIPF0000100 |
| RF00689 | MIR390 | MIPF0000101 |
| RF00690 | MIR408 | MIPF0000102 |
| RF00691 | mir-146 | MIPF0000103 |
| RF00692 | MIR171_2 | MIPF0000104 |
| RF00693 | mir-147 | MIPF0000105 |
| RF00694 | mir-137 | MIPF0000106 |
| RF00695 | MIR398 | MIPF0000107 |
| RF00696 | mir-203 | MIPF0000108 |
| RF00697 | mir-186 | MIPF0000109 |
| RF00698 | mir-489 | MIPF0000111 |
| RF00699 | mir-134 | MIPF0000112 |
| RF00700 | mir-375 | MIPF0000114 |
| RF00701 | mir-126 | MIPF0000115 |
| RF00702 | mir-182 | MIPF0000116 |
| RF00703 | mir-139 | MIPF0000117 |
| RF00704 | MIR397 | MIPF0000120 |
| RF00705 | mir-202 | MIPF0000121 |
| RF00706 | mir-263 | MIPF0000122 |
| RF00707 | mir-197 | MIPF0000123 |
| RF00708 | mir-450 | MIPF0000128 |
| RF00709 | mir-455 | MIPF0000129 |
| RF00710 | mir-44 | MIPF0000132 |
| RF00711 | mir-449 | MIPF0000133 |
| RF00712 | mir-460 | MIPF0000134 |
| RF00713 | mir-239 | MIPF0000135 |
| RF00714 | MIR535 | MIPF0000136 |
| RF00715 | mir-383 | MIPF0000137 |
| RF00716 | mir-3 | MIPF0000140 |
| RF00717 | mir-315 | MIPF0000141 |
| RF00718 | mir-431 | MIPF0000142 |
| RF00719 | mir-326 | MIPF0000143 |
| RF00720 | mir-317 | MIPF0000144 |
| RF00721 | MIR475 | MIPF0000145 |
| RF00722 | mir-451 | MIPF0000148 |
| RF00723 | mir-448 | MIPF0000149 |
| RF00724 | mir-282 | MIPF0000150 |
| RF00725 | mir-iab-4 | MIPF0000151 |
| RF00726 | mir-87 | MIPF0000152 |
| RF00727 | bantam | MIPF0000153 |
| RF00728 | mir-81 | MIPF0000154 |
| RF00729 | mir-278 | MIPF0000155 |
| RF00730 | mir-277 | MIPF0000156 |
| RF00731 | mir-155 | MIPF0000157 |
| RF00732 | mir-305 | MIPF0000158 |
| RF00733 | mir-296 | MIPF0000159 |
| RF00734 | mir-52 | MIPF0000160 |
| RF00735 | mir-367 | MIPF0000162 |
| RF00736 | mir-320 | MIPF0000163 |
| RF00737 | mir-322 | MIPF0000164 |
| RF00739 | MIR476 | MIPF0000166 |
| RF00740 | mir-370 | MIPF0000167 |
| RF00741 | mir-378 | MIPF0000168 |
| RF00742 | MIR162_1 | MIPF0000127 |
| RF00743 | mir-308 | MIPF0000171 |
| RF00744 | mir-361 | MIPF0000172 |
| RF00745 | mir-499 | MIPF0000173 |
| RF00746 | mir-454 | MIPF0000174 |
| RF00747 | mir-283 | MIPF0000175 |
| RF00748 | mir-433 | MIPF0000177 |
| RF00749 | mir-208 | MIPF0000178 |
| RF00750 | mir-458 | MIPF0000179 |
| RF00751 | mir-12 | MIPF0000181 |
| RF00752 | mir-14 | MIPF0000182 |
| RF00753 | mir-503 | MIPF0000183 |
| RF00754 | mir-279 | MIPF0000184 |
| RF00755 | mir-542 | MIPF0000185 |
| RF00756 | mir-299 | MIPF0000186 |
| RF00757 | mir-275 | MIPF0000187 |
| RF00758 | mir-346 | MIPF0000188 |
| RF00760 | mir-342 | MIPF0000190 |
| RF00761 | mir-340 | MIPF0000191 |
| RF00762 | mir-412 | MIPF0000192 |
| RF00763 | mir-339 | MIPF0000193 |
| RF00764 | mir-191 | MIPF0000194 |
| RF00765 | mir-337 | MIPF0000195 |
| RF00766 | mir-335 | MIPF0000196 |
| RF00767 | mir-150 | MIPF0000197 |
| RF00768 | MIR405 | MIPF0000198 |
| RF00769 | mir-331 | MIPF0000199 |
| RF00770 | mir-330 | MIPF0000200 |
| RF00771 | mir-185 | MIPF0000202 |
| RF00772 | mir-328 | MIPF0000203 |
| RF00773 | mir-298 | MIPF0000206 |
| RF00774 | mir-360 | MIPF0000210 |
| RF00775 | mir-432 | MIPF0000211 |
| RF00776 | mir-540 | MIPF0000212 |
| RF00777 | mir-541 | MIPF0000213 |
| RF00778 | MIR473 | MIPF0000214 |
| RF00779 | MIR474 | MIPF0000215 |
| RF00780 | MIR477 | MIPF0000216 |
| RF00781 | mir-505 | MIPF0000217 |
| RF00782 | MIR480 | MIPF0000218 |
| RF00783 | mir-484 | MIPF0000219 |
| RF00784 | mir-486 | MIPF0000220 |
| RF00785 | mir-90 | MIPF0000221 |
| RF00786 | mir-289 | MIPF0000222 |
| RF00787 | mir-288 | MIPF0000223 |
| RF00788 | mir-287 | MIPF0000224 |
| RF00789 | mir-286 | MIPF0000225 |
| RF00790 | mir-358 | MIPF0000226 |
| RF00791 | mir-357 | MIPF0000227 |
| RF00792 | mir-490 | MIPF0000229 |
| RF00793 | mir-497 | MIPF0000231 |
| RF00794 | mir-42 | MIPF0000233 |
| RF00795 | mir-43 | MIPF0000234 |
| RF00796 | mir-48 | MIPF0000235 |
| RF00797 | mir-355 | MIPF0000236 |
| RF00798 | mir-49 | MIPF0000237 |
| RF00799 | mir-354 | MIPF0000238 |
| RF00801 | mir-280 | MIPF0000240 |
| RF00802 | mir-207 | MIPF0000241 |
| RF00803 | mir-425 | MIPF0000242 |
| RF00804 | mir-240 | MIPF0000243 |
| RF00805 | mir-351 | MIPF0000244 |
| RF00806 | mir-350 | MIPF0000245 |
| RF00807 | mir-314 | MIPF0000246 |
| RF00808 | mir-86 | MIPF0000247 |
| RF00809 | mir-241 | MIPF0000248 |
| RF00810 | mir-85 | MIPF0000249 |
| RF00811 | mir-84 | MIPF0000250 |
| RF00812 | mir-83 | MIPF0000251 |
| RF00813 | mir-11 | MIPF0000252 |
| RF00814 | mir-316 | MIPF0000254 |
| RF00815 | mir-244 | MIPF0000256 |
| RF00816 | mir-245 | MIPF0000257 |
| RF00817 | mir-80 | MIPF0000258 |
| RF00818 | mir-318 | MIPF0000259 |
| RF00819 | mir-246 | MIPF0000260 |
| RF00820 | mir-248 | MIPF0000262 |
| RF00821 | mir-249 | MIPF0000263 |
| RF00822 | mir-274 | MIPF0000264 |
| RF00823 | lsy-6 | MIPF0000265 |
| RF00824 | mir-50 | MIPF0000266 |
| RF00825 | mir-344 | MIPF0000267 |
| RF00826 | mir-55 | MIPF0000270 |
| RF00827 | mir-77 | MIPF0000272 |
| RF00828 | mir-75 | MIPF0000273 |
| RF00829 | mir-149 | MIPF0000274 |
| RF00830 | mir-74 | MIPF0000275 |
| RF00831 | mir-73 | MIPF0000276 |
| RF00832 | mir-71 | MIPF0000278 |
| RF00833 | mir-70 | MIPF0000279 |
| RF00834 | mir-268 | MIPF0000280 |
| RF00835 | mir-58 | MIPF0000282 |
| RF00836 | mir-250 | MIPF0000283 |
| RF00837 | mir-251 | MIPF0000284 |
| RF00838 | mir-252 | MIPF0000285 |
| RF00839 | mir-452 | MIPF0000287 |
| RF00840 | mir-374 | MIPF0000288 |
| RF00841 | mir-384 | MIPF0000289 |
| RF00842 | MIR403 | MIPF0000290 |
| RF00843 | mir-228 | MIPF0000292 |
| RF00844 | mir-67 | MIPF0000293 |
| RF00845 | MIR158 | MIPF0000294 |
| RF00846 | mir-64 | MIPF0000295 |
| RF00847 | mir-62 | MIPF0000296 |
| RF00848 | mir-61 | MIPF0000297 |
| RF00849 | mir-60 | MIPF0000298 |
| RF00850 | mir-259 | MIPF0000299 |
| RF00851 | mir-230 | MIPF0000306 |
| RF00852 | mir-231 | MIPF0000307 |
| RF00853 | mir-304 | MIPF0000308 |
| RF00854 | mir-5 | MIPF0000309 |
| RF00855 | mir-254 | MIPF0000310 |
| RF00856 | mir-232 | MIPF0000311 |
| RF00857 | mir-233 | MIPF0000312 |
| RF00858 | mir-306 | MIPF0000313 |
| RF00859 | mir-234 | MIPF0000315 |
| RF00861 | mir-488 | MIPF0000318 |
| RF00862 | mir-491 | MIPF0000319 |
| RF00863 | mir-BART17 | MIPF0000321 |
| RF00864 | mir-BART20 | MIPF0000322 |
| RF00865 | MIR169_5 | MIPF0000323 |
| RF00866 | mir-BART3 | MIPF0000324 |
| RF00867 | mir-BART5 | MIPF0000326 |
| RF00868 | mir-BART15 | MIPF0000327 |
| RF00869 | mir-BART7 | MIPF0000328 |
| RF00870 | mir-423 | MIPF0000329 |
| RF00872 | mir-652 | MIPF0000333 |
| RF00873 | mir-550 | MIPF0000334 |
| RF00874 | mir-BART12 | MIPF0000335 |
| RF00875 | mir-692 | MIPF0000336 |
| RF00876 | mir-684 | MIPF0000337 |
| RF00877 | mir-592 | MIPF0000340 |
| RF00878 | mir-456 | MIPF0000341 |
| RF00879 | mir-615 | MIPF0000342 |
| RF00883 | MIR820 | MIPF0000348 |
| RF00884 | MIR815 | MIPF0000349 |
| RF00885 | MIR821 | MIPF0000350 |
| RF00886 | MIR807 | MIPF0000352 |
| RF00887 | mir-802 | MIPF0000353 |
| RF00888 | mir-770 | MIPF0000355 |
| RF00890 | mir-668 | MIPF0000357 |
| RF00891 | mir-671 | MIPF0000358 |
| RF00892 | mir-551 | MIPF0000360 |
| RF00893 | MIR854 | MIPF0000361 |
| RF00894 | mir-790 | MIPF0000362 |
| RF00895 | mir-786 | MIPF0000363 |
| RF00896 | mir-787 | MIPF0000364 |
| RF00897 | mir-675 | MIPF0000365 |
| RF00898 | mir-242 | MIPF0000366 |
| RF00899 | mir-235 | MIPF0000367 |
| RF00900 | mir-255 | MIPF0000368 |
| RF00901 | MIR845_1 | MIPF0000369 |
| RF00902 | mir-791 | MIPF0000370 |
| RF00903 | mir-359 | MIPF0000371 |
| RF00904 | mir-392 | MIPF0000372 |
| RF00905 | mir-789 | MIPF0000373 |
| RF00906 | MIR1122 | MIPF0000382 |
| RF00907 | mir-941 | MIPF0000387 |
| RF00908 | MIR529 | MIPF0000388 |
| RF00909 | mir-883 | MIPF0000389 |
| RF00910 | mir-873 | MIPF0000390 |
| RF00911 | mir-672 | MIPF0000391 |
| RF00912 | mir-877 | MIPF0000392 |
| RF00914 | mir-674 | MIPF0000394 |
| RF00915 | mir-760 | MIPF0000395 |
| RF00917 | mir-708 | MIPF0000397 |
| RF00918 | mir-872 | MIPF0000399 |
| RF00919 | mir-874 | MIPF0000401 |
| RF00920 | MIR444 | MIPF0000402 |
| RF00921 | mir-665 | MIPF0000404 |
| RF00922 | mir-673 | MIPF0000405 |
| RF00925 | MIR1027 | MIPF0000414 |
| RF00926 | MIR1151 | MIPF0000415 |
| RF00927 | mir-582 | MIPF0000417 |
| RF00928 | mir-590 | MIPF0000418 |
| RF00929 | mir-574 | MIPF0000419 |
| RF00931 | mir-879 | MIPF0000421 |
| RF00932 | mir-471 | MIPF0000423 |
| RF00933 | mir-875 | MIPF0000424 |
| RF00934 | mir-463 | MIPF0000425 |
| RF00935 | mir-876 | MIPF0000430 |
| RF00936 | mir-744 | MIPF0000431 |
| RF00937 | mir-653 | MIPF0000435 |
| RF00939 | mir-504 | MIPF0000437 |
| RF00940 | mir-327 | MIPF0000438 |
| RF00941 | mir-434 | MIPF0000439 |
| RF00942 | mir-1224 | MIPF0000440 |
| RF00943 | MIR824 | MIPF0000442 |
| RF00945 | mir-1226 | MIPF0000444 |
| RF00946 | mir-1225 | MIPF0000445 |
| RF00947 | mir-929 | MIPF0000447 |
| RF00948 | mir-996 | MIPF0000449 |
| RF00949 | mir-983 | MIPF0000450 |
| RF00950 | mir-927 | MIPF0000452 |
| RF00951 | mir-1302 | MIPF0000456 |
| RF00952 | mir-650 | MIPF0000457 |
| RF00953 | mir-1497 | MIPF0000458 |
| RF00954 | MIR1446 | MIPF0000459 |
| RF00955 | mir-1829 | MIPF0000460 |
| RF00956 | MIR1444 | MIPF0000461 |
| RF00957 | mir-663 | MIPF0000462 |
| RF00958 | mir-498 | MIPF0000463 |
| RF00959 | mir-612 | MIPF0000464 |
| RF00960 | mir-661 | MIPF0000465 |
| RF00961 | mir-581 | MIPF0000466 |
| RF00962 | mir-586 | MIPF0000467 |
| RF00963 | mir-642 | MIPF0000468 |
| RF00964 | mir-938 | MIPF0000469 |
| RF00965 | mir-549 | MIPF0000470 |
| RF00966 | mir-676 | MIPF0000471 |
| RF00967 | mir-281 | MIPF0000472 |
| RF00968 | mir-626 | MIPF0000474 |
| RF00969 | mir-556 | MIPF0000475 |
| RF00970 | mir-648 | MIPF0000476 |
| RF00971 | mir-578 | MIPF0000477 |
| RF00972 | mir-651 | MIPF0000478 |
| RF00973 | mir-597 | MIPF0000479 |
| RF00974 | mir-607 | MIPF0000480 |
| RF00975 | MIR845_2 | MIPF0000481 |
| RF00976 | mir-583 | MIPF0000482 |
| RF00977 | mir-600 | MIPF0000484 |
| RF00978 | mir-638 | MIPF0000486 |
| RF00979 | mir-553 | MIPF0000487 |
| RF00980 | mir-643 | MIPF0000488 |
| RF00981 | mir-939 | MIPF0000490 |
| RF00983 | mir-662 | MIPF0000492 |
| RF00984 | mir-576 | MIPF0000493 |
| RF00985 | mir-640 | MIPF0000494 |
| RF00986 | mir-920 | MIPF0000495 |
| RF00987 | mir-589 | MIPF0000496 |
| RF00988 | mir-657 | MIPF0000498 |
| RF00989 | mir-492 | MIPF0000499 |
| RF00990 | mir-552 | MIPF0000501 |
| RF00991 | mir-599 | MIPF0000502 |
| RF00992 | mir-593 | MIPF0000503 |
| RF00993 | mir-1473 | MIPF0000504 |
| RF00994 | mir-1255 | MIPF0000506 |
| RF00995 | mir-616 | MIPF0000507 |
| RF00996 | mir-631 | MIPF0000508 |
| RF00997 | mir-942 | MIPF0000511 |
| RF00998 | mir-562 | MIPF0000512 |
| RF00999 | mir-924 | MIPF0000513 |
| RF01000 | mir-580 | MIPF0000515 |
| RF01001 | mir-609 | MIPF0000516 |
| RF01002 | mir-936 | MIPF0000517 |
| RF01003 | mir-563 | MIPF0000519 |
| RF01004 | mir-557 | MIPF0000520 |
| RF01005 | MIR530 | MIPF0000521 |
| RF01006 | mir-601 | MIPF0000522 |
| RF01007 | mir-624 | MIPF0000523 |
| RF01008 | mir-636 | MIPF0000524 |
| RF01009 | mir-M7 | MIPF0000526 |
| RF01010 | mir-632 | MIPF0000527 |
| RF01011 | mir-605 | MIPF0000528 |
| RF01012 | mir-628 | MIPF0000529 |
| RF01013 | mir-577 | MIPF0000530 |
| RF01014 | mir-1306 | MIPF0000531 |
| RF01015 | mir-885 | MIPF0000532 |
| RF01016 | mir-584 | MIPF0000533 |
| RF01018 | mir-569 | MIPF0000535 |
| RF01019 | mir-922 | MIPF0000536 |
| RF01020 | mir-572 | MIPF0000537 |
| RF01021 | mir-558 | MIPF0000538 |
| RF01022 | mir-611 | MIPF0000539 |
| RF01023 | mir-940 | MIPF0000540 |
| RF01024 | mir-944 | MIPF0000541 |
| RF01025 | mir-934 | MIPF0000542 |
| RF01026 | MIR828 | MIPF0000544 |
| RF01027 | mir-765 | MIPF0000545 |
| RF01028 | mir-633 | MIPF0000546 |
| RF01029 | mir-649 | MIPF0000547 |
| RF01030 | mir-422 | MIPF0000548 |
| RF01031 | mir-639 | MIPF0000549 |
| RF01032 | mir-554 | MIPF0000551 |
| RF01033 | mir-767 | MIPF0000552 |
| RF01034 | mir-618 | MIPF0000553 |
| RF01035 | mir-887 | MIPF0000554 |
| RF01036 | mir-567 | MIPF0000555 |
| RF01037 | mir-644 | MIPF0000556 |
| RF01038 | mir-1307 | MIPF0000558 |
| RF01039 | mir-937 | MIPF0000560 |
| RF01040 | mir-573 | MIPF0000561 |
| RF01041 | mir-604 | MIPF0000562 |
| RF01042 | mir-891 | MIPF0000420 |
| RF01043 | MIR1023 | MIPF0000396 |
| RF01044 | mir-345 | MIPF0000189 |
| RF01045 | mir-544 | MIPF0000436 |
| RF01059 | mir-598 | MIPF0000393 |
| RF01061 | mir-548 | MIPF0000317 |
| RF01064 | mir-253 | MIPF0000286 |
| RF01117 | ciona-mir-92 | MIPF0000509 |
| RF01314 | mir-1227 | MIPF0000454 |
| RF01413 | miR-430 | MIPF0000003 |
| RF01895 | mir-193 | MIPF0000082 |
| RF01896 | mir-142 | MIPF0000084 |
| RF01897 | mir-188 | MIPF0000113 |
| RF01898 | mir-363 | MIPF0000138 |
| RF01899 | mir-2241 | MIPF0000821 |
| RF01900 | mir-2024 | MIPF0000697 |
| RF01901 | mir-284 | MIPF0000228 |
| RF01902 | MIR439 | MIPF0000092 |
| RF01903 | mir-500 | MIPF0000139 |
| RF01910 | mir-511 | MIPF0000130 |
| RF01911 | MIR2118 | MIPF0000745 |
| RF01912 | mir-2807 | MIPF0000826 |
| RF01913 | mir-2778 | MIPF0000827 |
| RF01914 | mir-932 | MIPF0000588 |
| RF01915 | mir-2238 | MIPF0000822 |
| RF01916 | mir-988 | MIPF0000724 |
| RF01917 | MIR2587 | MIPF0000835 |
| RF01918 | mir-1249 | MIPF0000667 |
| RF01919 | mir-1419 | MIPF0000570 |
| RF01920 | mir-764 | MIPF0000707 |
| RF01921 | mir-1296 | MIPF0000649 |
| RF01922 | mir-654 | MIPF0000409 |
| RF01923 | mir-711 | MIPF0000748 |
| RF01924 | mir-2774 | MIPF0000829 |
| RF01925 | MIR1428 | MIPF0000575 |
| RF01926 | mir-981 | MIPF0000710 |
| RF01927 | MIR1222 | MIPF0000410 |
| RF01936 | mir-63 | MIPF0000823 |
| RF01937 | mir-2780 | MIPF0000828 |
| RF01938 | mir-1251 | MIPF0000621 |
| RF01939 | mir-761 | MIPF0000709 |
| RF01940 | hvt-mir-H | MIPF0000783 |
| RF01941 | MIR1223 | MIPF0000383 |
| RF01943 | mir-999 | MIPF0000852 |
| RF01944 | mir-2518 | MIPF0000858 |
| RF01945 | mir-1388 | MIPF0000805 |
| RF01996 | mir-995 | MIPF0000895 |
| RF01997 | mir-969 | MIPF0000879 |
| RF02000 | MIR1846 | MIPF0000577 |
| RF02006 | mir-1253 | MIPF0000611 |
| RF02007 | mir-1237 | MIPF0000585 |
| RF02008 | mir-621 | MIPF0000631 |
| RF02009 | mir-987 | MIPF0000859 |
| RF02010 | mir-3180 | MIPF0000894 |
| RF02011 | mir-575 | MIPF0000586 |
| RF02014 | mir-1178 | MIPF0000601 |
| RF02015 | mir-1287 | MIPF0000725 |
| RF02016 | mir-1183 | MIPF0000668 |
| RF02017 | mir-1912 | MIPF0000768 |
| RF02018 | mir-1207 | MIPF0000596 |
| RF02019 | mir-1265 | MIPF0000688 |
| RF02020 | mir-25 | MIPF0000013 |
| RF02021 | mir-3179 | MIPF0000900 |
| RF02022 | mir-1275 | MIPF0000674 |
| RF02023 | mir-1208 | MIPF0000628 |
| RF02024 | mir-1180 | MIPF0000789 |
| RF02025 | mir-3017 | MIPF0001107 |
| RF02026 | mir-2833 | MIPF0000886 |
| RF02027 | MIR2907 | MIPF0000861 |
| RF02028 | mir-1827 | MIPF0000646 |
| RF02092 | mir-2970 | MIPF0001328 |
| RF02095 | mir-2985 | MIPF0001067 |
| RF02516 | mir-393 | MIPF0000083 |
| RF02518 | mir-2494 | MIPF0000975 |
| RF02520 | mir-965 | MIPF0000708 |
| RF03171 | mir-1012 | MIPF0001046 |
| RF03172 | mir-6012 | MIPF0001496 |
| RF03173 | mir-4504 | MIPF0001508 |
| RF03174 | mir-4803 | MIPF0001511 |
| RF03175 | MIR3454 | MIPF0001097 |
| RF03176 | MIR7729 | MIPF0001721 |
| RF03177 | mir-2067 | MIPF0001854 |
| RF03178 | mir-4515 | MIPF0001512 |
| RF03179 | mir-1182 | MIPF0000673 |
| RF03180 | mir-3126 | MIPF0001525 |
| RF03181 | mir-2755 | MIPF0001452 |
| RF03182 | MIR6032 | MIPF0002003 |
| RF03183 | MIR5516 | MIPF0001595 |
| RF03184 | mir-1250 | MIPF0000696 |
| RF03185 | mir-3193 | MIPF0001401 |
| RF03186 | mir-3689 | MIPF0001144 |
| RF03187 | MIR1512 | MIPF0001291 |
| RF03188 | mir-2843 | MIPF0001044 |
| RF03189 | mir-1175 | MIPF0000715 |
| RF03190 | mir-4857 | MIPF0001842 |
| RF03191 | mir-1913 | MIPF0001015 |
| RF03192 | mir-6794 | MIPF0001726 |
| RF03193 | mir-2941 | MIPF0000842 |
| RF03194 | mir-1272 | MIPF0000645 |
| RF03195 | mir-3934 | MIPF0001584 |
| RF03196 | mir-6790 | MIPF0001811 |
| RF03197 | mir-4887 | MIPF0001841 |
| RF03198 | mir-7385 | MIPF0001688 |
| RF03199 | MIR5271 | MIPF0001281 |
| RF03200 | mir-BART13 | MIPF0001051 |
| RF03201 | mir-617 | MIPF0000672 |
| RF03202 | mir-555 | MIPF0000583 |
| RF03203 | mir-3070 | MIPF0001172 |
| RF03204 | mir-4510 | MIPF0001467 |
| RF03205 | mir-1898 | MIPF0000786 |
| RF03206 | mir-3102 | MIPF0001469 |
| RF03207 | mir-3960 | MIPF0001271 |
| RF03208 | mir-1781 | MIPF0001583 |
| RF03209 | MIR9657 | MIPF0001955 |
| RF03210 | mir-7359 | MIPF0001650 |
| RF03211 | mir-666 | MIPF0000782 |
| RF03212 | mir-2788 | MIPF0001204 |
| RF03213 | mir-5885 | MIPF0001404 |
| RF03214 | mir-769 | MIPF0000727 |
| RF03215 | mir-5360 | MIPF0001723 |
| RF03216 | mir-BART10 | MIPF0000850 |
| RF03217 | mir-3167 | MIPF0001823 |
| RF03218 | mir-3015 | MIPF0001111 |
| RF03219 | mir-602 | MIPF0000767 |
| RF03220 | mir-2582 | MIPF0000870 |
| RF03221 | mir-6715 | MIPF0001708 |
| RF03222 | mir-4900 | MIPF0001224 |
| RF03223 | mir-2209 | MIPF0000787 |
| RF03224 | MIR9560 | MIPF0002116 |
| RF03225 | mir-2763 | MIPF0001437 |
| RF03226 | mir-8196 | MIPF0002057 |
| RF03227 | mir-2528 | MIPF0001059 |
| RF03228 | mir-1471 | MIPF0001069 |
| RF03229 | mir-1297 | MIPF0000584 |
| RF03230 | mir-967 | MIPF0001096 |
| RF03231 | mir-4523 | MIPF0002104 |
| RF03232 | mir-6526 | MIPF0001431 |
| RF03233 | hvt-mir-H16 | MIPF0000773 |
| RF03234 | mir-2813 | MIPF0001043 |
| RF03235 | mir-2543 | MIPF0000856 |
| RF03236 | mir-1206 | MIPF0000634 |
| RF03237 | MIR6425 | MIPF0001386 |
| RF03238 | mir-1542 | MIPF0000485 |
| RF03239 | mir-963 | MIPF0001035 |
| RF03240 | MIR5534 | MIPF0001324 |
| RF03241 | mir-1789 | MIPF0001636 |
| RF03242 | mir-1397 | MIPF0000642 |
| RF03243 | mir-2731 | MIPF0000795 |
| RF03244 | mir-4798 | MIPF0001574 |
| RF03246 | MIR1515 | MIPF0001591 |
| RF03247 | mir-1324 | MIPF0000589 |
| RF03248 | MIR9772 | MIPF0001934 |
| RF03249 | mir-2114 | MIPF0001464 |
| RF03251 | mir-4446 | MIPF0001385 |
| RF03252 | mir-678 | MIPF0000774 |
| RF03253 | mir-4654 | MIPF0001507 |
| RF03254 | mir-9460 | MIPF0001910 |
| RF03255 | mir-2188 | MIPF0000812 |
| RF03256 | mir-1286 | MIPF0000682 |
| RF03257 | mir-4427 | MIPF0001640 |
| RF03258 | mir-4 | MIPF0000305 |
| RF03259 | MIR1514 | MIPF0000694 |
| RF03260 | mir-8229 | MIPF0001992 |
| RF03261 | mir-1759 | MIPF0001565 |
| RF03262 | mir-1181 | MIPF0000665 |
| RF03263 | MIR861 | MIPF0001190 |
| RF03264 | MIR1024 | MIPF0000406 |
| RF03265 | mir-1460 | MIPF0001502 |
| RF03266 | mir-2943 | MIPF0001056 |
| RF03267 | mir-645 | MIPF0000657 |
| RF03268 | mir-1911 | MIPF0001787 |
| RF03269 | mir-5697 | MIPF0001748 |
| RF03270 | MIR3712 | MIPF0001625 |
| RF03271 | mir-1247 | MIPF0000669 |
| RF03272 | mir-2187 | MIPF0001770 |
| RF03274 | mir-1238 | MIPF0000941 |
| RF03275 | mir-2542 | MIPF0000889 |
| RF03276 | mir-2232 | MIPF0001054 |
| RF03277 | mir-5886 | MIPF0001638 |
| RF03278 | mir-980 | MIPF0000890 |
| RF03279 | mir-1193 | MIPF0000714 |
| RF03280 | mir-7583 | MIPF0001676 |
| RF03281 | mir-596 | MIPF0000808 |
| RF03283 | mir-1010 | MIPF0000866 |
| RF03284 | mir-4637 | MIPF0001586 |
| RF03285 | mir-564 | MIPF0000612 |
| RF03286 | MIR6146 | MIPF0001562 |
| RF03287 | mir-5904 | MIPF0001444 |
| RF03288 | mir-3085 | MIPF0001193 |
| RF03289 | mir-1205 | MIPF0000609 |
| RF03290 | mir-9428 | MIPF0001958 |
| RF03291 | mir-591 | MIPF0000641 |
| RF03292 | mir-1301 | MIPF0000742 |
| RF03293 | mir-4672 | MIPF0001551 |
| RF03294 | mir-H3 | MIPF0000717 |
| RF03295 | mir-7594 | MIPF0001674 |
| RF03296 | MIR8788 | MIPF0001903 |
| RF03297 | MIR1033 | MIPF0000385 |
| RF03298 | mir-4488 | MIPF0001623 |
| RF03299 | mir-1000 | MIPF0000701 |
| RF03300 | mir-3617 | MIPF0001477 |
| RF03301 | mir-3154 | MIPF0001387 |
| RF03302 | mir-6133 | MIPF0001415 |
| RF03303 | mir-973 | MIPF0001970 |
| RF03304 | mir-1744 | MIPF0001589 |
| RF03305 | mir-3338 | MIPF0001449 |
| RF03306 | mir-3923 | MIPF0001527 |
| RF03307 | mir-1825 | MIPF0000670 |
| RF03308 | MIR848 | MIPF0001140 |
| RF03309 | mir-4791 | MIPF0001529 |
| RF03310 | mir-1544 | MIPF0001645 |
| RF03311 | MIR169 | MIPF0000832 |
| RF03312 | mir-880 | MIPF0000433 |
| RF03313 | mir-1468 | MIPF0000777 |
| RF03314 | mir-1908 | MIPF0001021 |
| RF03315 | mir-723 | MIPF0001831 |
| RF03316 | mir-1263 | MIPF0000687 |
| RF03317 | mir-4874 | MIPF0001843 |
| RF03318 | mir-2208 | MIPF0000772 |
| RF03319 | mir-989 | MIPF0000885 |
| RF03320 | mir-1743 | MIPF0001544 |
| RF03321 | mir-1006 | MIPF0000873 |
| RF03322 | mir-1641 | MIPF0001533 |
| RF03323 | mir-3613 | MIPF0001411 |
| RF03324 | mir-1288 | MIPF0000660 |
| RF03325 | mir-4872 | MIPF0001205 |
| RF03326 | mir-312 | MIPF0000573 |
| RF03327 | mir-1266 | MIPF0000615 |
| RF03328 | mir-606 | MIPF0000780 |
| RF03329 | mir-4743 | MIPF0001384 |
| RF03330 | mir-750 | MIPF0000796 |
| RF03331 | mir-247 | MIPF0001019 |
| RF03332 | mir-2237 | MIPF0000883 |
| RF03333 | mir-2820 | MIPF0001034 |
| RF03334 | MIR391 | MIPF0001151 |
| RF03335 | MIR2950 | MIPF0001121 |
| RF03336 | mir-1322 | MIPF0000663 |
| RF03337 | mir-6505 | MIPF0001805 |
| RF03338 | MIR4416 | MIPF0001389 |
| RF03339 | mir-3047 | MIPF0001196 |
| RF03340 | mir-6511 | MIPF0001382 |
| RF03341 | mir-943 | MIPF0000690 |
| RF03342 | mir-1467 | MIPF0001416 |
| RF03343 | mir-3347 | MIPF0001022 |
| RF03344 | mir-5365 | MIPF0001363 |
| RF03345 | MIR3444 | MIPF0001177 |
| RF03346 | mir-881 | MIPF0000429 |
| RF03347 | mir-2816 | MIPF0000884 |
| RF03348 | mir-667 | MIPF0000794 |
| RF03349 | mir-3151 | MIPF0001394 |
| RF03350 | mir-3851 | MIPF0001201 |
| RF03351 | MIR6020 | MIPF0001610 |
| RF03352 | mir-4869 | MIPF0001829 |
| RF03353 | MIR5291 | MIPF0001293 |
| RF03354 | mir-459 | MIPF0001322 |
| RF03355 | mir-2008 | MIPF0000729 |
| RF03356 | mir-4782 | MIPF0001546 |
| RF03357 | mir-1842 | MIPF0000788 |
| RF03358 | mir-634 | MIPF0000600 |
| RF03359 | mir-1729 | MIPF0000816 |
| RF03360 | mir-3156 | MIPF0000891 |
| RF03361 | mir-3136 | MIPF0001575 |
| RF03362 | mir-9436 | MIPF0002111 |
| RF03363 | mir-6129 | MIPF0001423 |
| RF03364 | mir-427 | MIPF0000441 |
| RF03365 | mir-1236 | MIPF0000689 |
| RF03366 | mir-3122 | MIPF0001549 |
| RF03367 | mir-975 | MIPF0001038 |
| RF03368 | mir-1822 | MIPF0001009 |
| RF03369 | mir-2217 | MIPF0001912 |
| RF03370 | mir-2566 | MIPF0000880 |
| RF03371 | mir-4788 | MIPF0001573 |
| RF03372 | mir-5918 | MIPF0001395 |
| RF03373 | mir-3170 | MIPF0001637 |
| RF03374 | mir-2001 | MIPF0000711 |
| RF03375 | MIR1094 | MIPF0000400 |
| RF03376 | mir-3075 | MIPF0001489 |
| RF03377 | mir-960 | MIPF0000897 |
| RF03378 | mir-7398 | MIPF0001643 |
| RF03379 | mir-4667 | MIPF0001390 |
| RF03380 | mir-9441 | MIPF0002092 |
| RF03381 | mir-2796 | MIPF0001057 |
| RF03382 | mir-635 | MIPF0000662 |
| RF03383 | mir-1976 | MIPF0001633 |
| RF03384 | mir-7180 | MIPF0002036 |
| RF03385 | mir-614 | MIPF0000647 |
| RF03386 | mir-1994 | MIPF0000743 |
| RF03387 | mir-1902 | MIPF0000791 |
| RF03388 | mir-9418 | MIPF0002040 |
| RF03389 | mir-4738 | MIPF0001521 |
| RF03390 | mir-2450 | MIPF0000758 |
| RF03391 | mir-588 | MIPF0000681 |
| RF03392 | mir-3943 | MIPF0001400 |
| RF03393 | mir-2849 | MIPF0000876 |
| RF03394 | mir-2574 | MIPF0001029 |
| RF03395 | mir-718 | MIPF0000721 |
| RF03396 | mir-2814 | MIPF0001070 |
| RF03397 | mir-1234 | MIPF0000593 |
| RF03398 | mir-3552 | MIPF0002093 |
| RF03399 | mir-986 | MIPF0000860 |
| RF03400 | mir-728 | MIPF0001670 |
| RF03401 | mir-1323 | MIPF0000617 |
| RF03402 | mir-957 | MIPF0000723 |
| RF03403 | mir-5366 | MIPF0001738 |
| RF03404 | mir-1298 | MIPF0000598 |
| RF03405 | mir-3200 | MIPF0001441 |
| RF03406 | mir-3099 | MIPF0001559 |
| RF03407 | MIR531 | MIPF0000607 |
| RF03408 | mir-5890 | MIPF0001378 |
| RF03409 | mir-646 | MIPF0000605 |
| RF03410 | mir-1891 | MIPF0000857 |
| RF03411 | mir-2856 | MIPF0001071 |
| RF03412 | mir-1915 | MIPF0001037 |
| RF03413 | MIR904 | MIPF0000379 |
| RF03414 | mir-670 | MIPF0000734 |
| RF03416 | mir-7383 | MIPF0001662 |
| RF03417 | mir-343 | MIPF0000411 |
| RF03418 | mir-3198 | MIPF0001216 |
| RF03419 | mir-998 | MIPF0000952 |
| RF03420 | mir-1200 | MIPF0001036 |
| RF03421 | mir-2558 | MIPF0000817 |
| RF03422 | mir-637 | MIPF0000597 |
| RF03423 | mir-962 | MIPF0001011 |
| RF03424 | mir-4856 | MIPF0001847 |
| RF03425 | mir-1292 | MIPF0000675 |
| RF03426 | mir-1294 | MIPF0000603 |
| RF03427 | mir-1005 | MIPF0000943 |
| RF03428 | mir-6134 | MIPF0001445 |
| RF03429 | mir-4451 | MIPF0001582 |
| RF03430 | MIR5294 | MIPF0001303 |
| RF03431 | mir-4657 | MIPF0002009 |
| RF03432 | mir-7331 | MIPF0001736 |
| RF03433 | mir-2576 | MIPF0001066 |
| RF03434 | mir-1246 | MIPF0000627 |
| RF03435 | mir-726 | MIPF0001344 |
| RF03436 | mir-759 | MIPF0000706 |
| RF03437 | mir-2392 | MIPF0001614 |
| RF03438 | mir-1956 | MIPF0001470 |
| RF03439 | mir-1791 | MIPF0001396 |
| RF03440 | MIR4414 | MIPF0001299 |
| RF03441 | mir-613 | MIPF0000590 |
| RF03442 | mir-4003 | MIPF0000840 |
| RF03443 | mir-2131 | MIPF0001631 |
| RF03444 | MIR5635 | MIPF0001283 |
| RF03445 | mir-7552 | MIPF0001916 |
| RF03446 | mir-2954 | MIPF0001032 |
| RF03447 | mir-1231 | MIPF0001309 |
| RF03448 | mir-6131 | MIPF0001412 |
| RF03449 | mir-6827 | MIPF0001747 |
| RF03450 | MIR1513 | MIPF0001360 |
| RF03451 | mir-3188 | MIPF0001488 |
| RF03452 | mir-3121 | MIPF0001417 |
| RF03453 | mir-1276 | MIPF0000652 |
| RF03454 | mir-1267 | MIPF0000640 |
| RF03455 | mir-9847 | MIPF0002065 |
| RF03456 | mir-724 | MIPF0001616 |
| RF03457 | MIR173 | MIPF0001175 |
| RF03458 | mir-766 | MIPF0000677 |
| RF03459 | mir-1890 | MIPF0000848 |
| RF03460 | mir-561 | MIPF0000623 |
| RF03461 | MIR842 | MIPF0001158 |
| RF03462 | mir-4677 | MIPF0001848 |
| RF03463 | mir-3036 | MIPF0001098 |
| RF03464 | mir-465 | MIPF0000384 |
| RF03465 | MIR9666 | MIPF0001930 |
| RF03466 | mir-1184 | MIPF0000592 |
| RF03467 | mir-2851 | MIPF0001014 |
| RF03468 | mir-2830 | MIPF0001052 |
| RF03469 | mir-1179 | MIPF0000632 |
| RF03470 | mir-571 | MIPF0000757 |
| RF03471 | mir-745 | MIPF0001267 |
| RF03472 | mir-623 | MIPF0000798 |
| RF03473 | mir-8813 | MIPF0002069 |
| RF03474 | mir-4766 | MIPF0001764 |
| RF03475 | mir-2387 | MIPF0001986 |
| RF03476 | mir-7360 | MIPF0001649 |
| RF03477 | MIR862_2 | MIPF0001295 |
| RF03478 | mir-630 | MIPF0000653 |
| RF03479 | mir-324 | MIPF0000165 |
| RF03480 | mir-1284 | MIPF0000604 |
| RF03481 | mir-9014 | MIPF0002031 |
| RF03482 | mir-4774 | MIPF0001578 |
| RF03483 | MIR319 | MIPF0001104 |
| RF03484 | mir-K12-10 | MIPF0000261 |
| RF03485 | mir-4000 | MIPF0000867 |
| RF03486 | mir-3940 | MIPF0001599 |
| RF03487 | mir-4423 | MIPF0001587 |
| RF03488 | mir-2366 | MIPF0001403 |
| RF03489 | mir-4796 | MIPF0001402 |
| RF03490 | mir-1426 | MIPF0000692 |
| RF03491 | mir-3146 | MIPF0001626 |
| RF03492 | mir-878 | MIPF0000434 |
| RF03493 | mir-1281 | MIPF0000695 |
| RF03494 | mir-4680 | MIPF0001988 |
| RF03495 | mir-968 | MIPF0000865 |
| RF03496 | MIR2086 | MIPF0001302 |
| RF03497 | MIR5038 | MIPF0001368 |
| RF03498 | mir-3145 | MIPF0001427 |
| RF03499 | mir-702 | MIPF0001163 |
| RF03500 | mir-1262 | MIPF0000595 |
| RF03501 | mir-3140 | MIPF0001615 |
| RF03502 | mir-1914 | MIPF0001040 |
| RF03503 | mir-547 | MIPF0000779 |
| RF03504 | mir-9461 | MIPF0002000 |
| RF03505 | mir-9128 | MIPF0002044 |
| RF03506 | mir-587 | MIPF0000563 |
| RF03507 | mir-4429 | MIPF0001474 |
| RF03508 | mir-970 | MIPF0000700 |
| RF03509 | mir-4660 | MIPF0001460 |
| RF03510 | mir-3660 | MIPF0001397 |
| RF03511 | mir-5444 | MIPF0001358 |
| RF03512 | mir-3479 | MIPF0001629 |
| RF03514 | mir-6128 | MIPF0001442 |
| RF03516 | MIR5229 | MIPF0001323 |
| RF03517 | MIR5225 | MIPF0001388 |
| RF03518 | mir-1893 | MIPF0001812 |
| RF03519 | mir-238 | MIPF0000862 |
| RF03520 | mir-3059 | MIPF0001766 |
| RF03521 | mir-23c | MIPF0001429 |
| RF03522 | mir-4850 | MIPF0002086 |
| RF03523 | mir-4703 | MIPF0001759 |
| RF03524 | mir-3174 | MIPF0001569 |
| RF03525 | mir-1343 | MIPF0001206 |
| RF03526 | mir-7177 | MIPF0002020 |
| RF03527 | mir-3072 | MIPF0001410 |
| RF03528 | mir-1264 | MIPF0000098 |
| RF03548 | mir-302_2 | MIPF0000658 |
| RF03549 | mir-15_2 | MIPF0001828 |
| RF03550 | mir-31_2 | MIPF0001803 |
| RF03552 | mir-43_2 | MIPF0001285 |
| RF03553 | mir-190_2 | MIPF0001853 |
| RF03554 | mir-92_2 | MIPF0001099 |
| RF03555 | mir-2_2 | MIPF0001838 |
| RF03556 | mir-559 | MIPF0000656 |
| RF03557 | MIR6019 | MIPF0001486 |
| RF03558 | mir-629 | MIPF0001555 |
| RF03559 | mir-8303 | MIPF0002121 |
| RF03560 | mir-3926 | MIPF0001118 |
| RF03561 | mir-3533 | MIPF0002063 |
| RF03562 | mir-2004 | MIPF0002042 |
| RF03563 | mir-BART16 | MIPF0000991 |
| RF03564 | mir-9416 | MIPF0002105 |
| RF03565 | mir-1282 | MIPF0000587 |
| RF03566 | mir-4875 | MIPF0001222 |
| RF03567 | MIR8024 | MIPF0001789 |
| RF03568 | mir-3204 | MIPF0000908 |
| RF03569 | mir-7973 | MIPF0001693 |
| RF03570 | mir-8286 | MIPF0001964 |
| RF03571 | mir-734 | MIPF0001543 |
| RF03572 | MIR2109 | MIPF0001000 |
| RF03573 | mir-1954 | MIPF0001275 |
| RF03574 | mir-4847 | MIPF0002100 |
| RF03575 | mir-rL1-14 | MIPF0000993 |
| RF03576 | mir-4435 | MIPF0001220 |
| RF03577 | mir-9193 | MIPF0002089 |
| RF03578 | mir-3910 | MIPF0001148 |
| RF03579 | mir-2058 | MIPF0001793 |
| RF03580 | mir-928 | MIPF0000950 |
| RF03581 | mir-4878 | MIPF0001827 |
| RF03582 | mir-m22-1 | MIPF0000451 |
| RF03583 | MIR781 | MIPF0001137 |
| RF03584 | mir-2290 | MIPF0001973 |
| RF03585 | mir-3938 | MIPF0001520 |
| RF03586 | mir-5132 | MIPF0002061 |
| RF03587 | mir-1202 | MIPF0000613 |
| RF03588 | mir-2319 | MIPF0000790 |
| RF03589 | mir-5583 | MIPF0001356 |
| RF03590 | mir-2009 | MIPF0001923 |
| RF03591 | mir-3117 | MIPF0001506 |
| RF03592 | mir-2567 | MIPF0000934 |
| RF03593 | mir-2331 | MIPF0001951 |
| RF03594 | mir-3817 | MIPF0001217 |
| RF03595 | mir-3569 | MIPF0001797 |
| RF03596 | mir-5880 | MIPF0001483 |
| RF03597 | MIR6438 | MIPF0001538 |
| RF03598 | MIR6024 | MIPF0001459 |
| RF03599 | MIR1883 | MIPF0000683 |
| RF03600 | MIR3627 | MIPF0001471 |
| RF03601 | MIR7993 | MIPF0001663 |
| RF03602 | mir-8250 | MIPF0002067 |
| RF03603 | mir-4899 | MIPF0001837 |
| RF03604 | mir-5728 | MIPF0001558 |
| RF03605 | MIR4221 | MIPF0001128 |
| RF03606 | mir-2071 | MIPF0001681 |
| RF03607 | mir-9447 | MIPF0001994 |
| RF03608 | mir-9440 | MIPF0001915 |
| RF03609 | mir-5470 | MIPF0001967 |
| RF03610 | mir-H7 | MIPF0000929 |
| RF03611 | mir-7147 | MIPF0001705 |
| RF03612 | mir-608 | MIPF0000972 |
| RF03613 | MIR6234 | MIPF0001642 |
| RF03614 | mir-3431 | MIPF0002046 |
| RF03615 | mir-4877 | MIPF0001814 |
| RF03616 | MIR1888 | MIPF0001333 |
| RF03617 | MIR8757 | MIPF0002074 |
| RF03618 | mir-5592 | MIPF0001366 |
| RF03619 | mir-5729 | MIPF0001473 |
| RF03620 | mir-1233 | MIPF0000578 |
| RF03621 | MIR4245 | MIPF0001495 |
| RF03622 | mir-731 | MIPF0001354 |
| RF03623 | mir-1252 | MIPF0000968 |
| RF03624 | mir-5345 | MIPF0001357 |
| RF03625 | MIR6152 | MIPF0001607 |
| RF03626 | mir-9791 | MIPF0002118 |
| RF03627 | mir-9168 | MIPF0002095 |
| RF03628 | mir-2355 | MIPF0000938 |
| RF03629 | mir-641 | MIPF0000679 |
| RF03630 | mir-2818 | MIPF0001003 |
| RF03631 | MIR3440 | MIPF0001127 |
| RF03632 | MIR1882 | MIPF0000571 |
| RF03633 | mir-5882 | MIPF0001418 |
| RF03634 | mir-3524 | MIPF0001215 |
| RF03635 | mir-2066 | MIPF0001819 |
| RF03636 | MIR4376 | MIPF0001809 |
| RF03637 | mir-5879 | MIPF0001472 |
| RF03638 | mir-1537 | MIPF0000917 |
| RF03639 | mir-619 | MIPF0000557 |
| RF03640 | mir-3747 | MIPF0001223 |
| RF03641 | mir-991 | MIPF0000957 |
| RF03642 | MIR5743 | MIPF0001572 |
| RF03643 | mir-2056 | MIPF0001734 |
| RF03644 | mir-2064 | MIPF0001727 |
| RF03645 | mir-9080 | MIPF0001982 |
| RF03646 | MIR9776 | MIPF0001990 |
| RF03647 | mir-7640 | MIPF0001783 |
| RF03648 | mir-9413 | MIPF0002079 |
| RF03649 | MIR5643 | MIPF0001348 |
| RF03650 | mir-1290 | MIPF0000610 |
| RF03651 | mir-2070 | MIPF0001796 |
| RF03652 | mir-2329 | MIPF0000785 |
| RF03653 | mir-971 | MIPF0000995 |
| RF03654 | MIR4239 | MIPF0001164 |
| RF03655 | MIR1887 | MIPF0001125 |
| RF03656 | mir-2432 | MIPF0002055 |
| RF03657 | MIR6145 | MIPF0001620 |
| RF03658 | mir-2300 | MIPF0000770 |
| RF03659 | MIR2871 | MIPF0000986 |
| RF03660 | mir-4018 | MIPF0001259 |
| RF03661 | mir-933 | MIPF0000505 |
| RF03662 | mir-2060 | MIPF0001654 |
| RF03663 | mir-5911 | MIPF0001407 |
| RF03664 | MIR6288 | MIPF0001696 |
| RF03665 | MIR4359 | MIPF0001181 |
| RF03666 | MIR7724 | MIPF0001802 |
| RF03667 | MIR1031 | MIPF0000416 |
| RF03668 | mir-1788 | MIPF0000792 |
| RF03669 | mir-2767 | MIPF0001446 |
| RF03670 | MIR3932 | MIPF0001159 |
| RF03671 | MIR3522 | MIPF0000936 |
| RF03672 | MIR840 | MIPF0001129 |
| RF03673 | mir-2822 | MIPF0000970 |
| RF03674 | MIR5387 | MIPF0001305 |
| RF03675 | mir-1001 | MIPF0001048 |
| RF03676 | MIR5148 | MIPF0001298 |
| RF03677 | mir-1304 | MIPF0001064 |
| RF03678 | mir-3956 | MIPF0001577 |
| RF03679 | MIR2084 | MIPF0000809 |
| RF03680 | mir-9465 | MIPF0002107 |
| RF03681 | mir-1295 | MIPF0000676 |
| RF03682 | mir-3912 | MIPF0001601 |
| RF03683 | mir-462 | MIPF0001755 |
| RF03684 | mir-1990 | MIPF0000718 |
| RF03685 | MIR9677 | MIPF0001925 |
| RF03686 | mir-9424 | MIPF0002037 |
| RF03687 | mir-2011 | MIPF0000739 |
| RF03688 | mir-3045 | MIPF0001120 |
| RF03689 | mir-5892 | MIPF0001539 |
| RF03690 | mir-9058 | MIPF0002028 |
| RF03691 | mir-2765 | MIPF0001008 |
| RF03692 | MIR827 | MIPF0000726 |
| RF03693 | MIR1919 | MIPF0000582 |
| RF03694 | mir-266 | MIPF0000281 |
| RF03695 | MIR6151 | MIPF0001374 |
| RF03696 | mir-4904 | MIPF0001786 |
| RF03697 | mir-722 | MIPF0001612 |
| RF03698 | mir-2861 | MIPF0000963 |
| RF03699 | mir-729 | MIPF0001307 |
| RF03700 | mir-1923 | MIPF0000930 |
| RF03701 | mir-4041 | MIPF0001266 |
| RF03702 | MIR851 | MIPF0001168 |
| RF03703 | mir-3622 | MIPF0001179 |
| RF03704 | MIR4380 | MIPF0001117 |
| RF03705 | mir-1278 | MIPF0000591 |
| RF03706 | mir-2253 | MIPF0000915 |
| RF03707 | mir-1892 | MIPF0000799 |
| RF03709 | MIR1878 | MIPF0001218 |
| RF03710 | mir-1451 | MIPF0001049 |
| RF03711 | mir-784 | MIPF0000999 |
| RF03712 | MIR6450 | MIPF0001528 |
| RF03713 | mir-1756 | MIPF0000639 |
| RF03714 | MIR774 | MIPF0001101 |
| RF03715 | mir-977 | MIPF0000931 |
| RF03716 | mir-7131 | MIPF0001675 |
| RF03717 | mir-3119 | MIPF0000971 |
| RF03718 | mir-2804 | MIPF0000851 |
| RF03719 | mir-3914 | MIPF0001169 |
| RF03720 | MIR4228 | MIPF0001185 |
| RF03721 | mir-3478 | MIPF0002075 |
| RF03722 | MIR4378 | MIPF0001192 |
| RF03723 | MIR7125 | MIPF0001851 |
| RF03724 | mir-1013 | MIPF0000985 |
| RF03725 | mir-7094 | MIPF0001704 |
| RF03726 | mir-m21-1 | MIPF0000453 |
| RF03727 | mir-3477 | MIPF0001227 |
| RF03728 | MIR1511 | MIPF0001743 |
| RF03729 | mir-2363 | MIPF0000806 |
| RF03730 | mir-7615 | MIPF0001729 |
| RF03731 | mir-7371 | MIPF0001652 |
| RF03732 | mir-2057 | MIPF0001745 |
| RF03733 | mir-BART14 | MIPF0000959 |
| RF03734 | mir-4773 | MIPF0001250 |
| RF03735 | mir-1502 | MIPF0001213 |
| RF03736 | mir-3130 | MIPF0000845 |
| RF03737 | mir-8315 | MIPF0002039 |
| RF03738 | mir-2320 | MIPF0001253 |
| RF03739 | mir-4529 | MIPF0001451 |
| RF03740 | mir-921 | MIPF0000982 |
| RF03741 | mir-325 | MIPF0000147 |
| RF03742 | mir-2162 | MIPF0001950 |
| RF03743 | MIR7696 | MIPF0001669 |
| RF03744 | MIR3704 | MIPF0001425 |
| RF03745 | mir-2063 | MIPF0001752 |
| RF03746 | mir-2318 | MIPF0001968 |
| RF03747 | mir-4536 | MIPF0001319 |
| RF03748 | mir-2772 | MIPF0000906 |
| RF03749 | MIR2617 | MIPF0000907 |
| RF03750 | mir-2489 | MIPF0000953 |
| RF03751 | mir-7445 | MIPF0001857 |
| RF03752 | MIR5292 | MIPF0001329 |
| RF03753 | mir-1986 | MIPF0000741 |
| RF03754 | mir-3116 | MIPF0001002 |
| RF03755 | mir-4005 | MIPF0001007 |
| RF03756 | mir-4891 | MIPF0001856 |
| RF03757 | mir-6536 | MIPF0001479 |
| RF03758 | mir-2756 | MIPF0001391 |
| RF03759 | MIR8703 | MIPF0001959 |
| RF03760 | mir-2041 | MIPF0001913 |
| RF03761 | mir-5897 | MIPF0001517 |
| RF03762 | MIR859 | MIPF0001191 |
| RF03763 | mir-2568 | MIPF0001004 |
| RF03764 | mir-647 | MIPF0000939 |
| RF03765 | mir-2059 | MIPF0001683 |
| RF03766 | mir-3155 | MIPF0001242 |
| RF03767 | mir-3432 | MIPF0001187 |
| RF03768 | mir-1329 | MIPF0000635 |
| RF03769 | mir-3913 | MIPF0001134 |
| RF03770 | mir-125 | MIPF0000733 |
| RF03771 | mir-341 | MIPF0000269 |
| RF03772 | mir-4867 | MIPF0001707 |
| RF03773 | mir-978 | MIPF0000926 |
| RF03774 | mir-2110 | MIPF0001399 |
| RF03775 | mir-7578 | MIPF0001810 |
| RF03776 | mir-2068 | MIPF0001700 |
| RF03777 | MIR533 | MIPF0000356 |
| RF03778 | mir-483 | MIPF0000180 |
| RF03779 | mir-1805 | MIPF0000947 |
| RF03780 | mir-3389 | MIPF0001581 |
| RF03781 | mir-4524 | MIPF0001330 |
| RF03782 | mir-3661 | MIPF0001530 |
| RF03783 | mir-4854 | MIPF0001908 |
| RF03784 | mir-2240 | MIPF0000838 |
| RF03785 | mir-974 | MIPF0002083 |
| RF03786 | mir-9449 | MIPF0001976 |
| RF03787 | mir-2010 | MIPF0002019 |
| RF03788 | MIR5304 | MIPF0001713 |
| RF03789 | MIR5269 | MIPF0001370 |
| RF03790 | MIR8008 | MIPF0001836 |
| RF03791 | mir-4526 | MIPF0001545 |
| RF03792 | MIR6462 | MIPF0001393 |
| RF03793 | MIR810 | MIPF0000497 |
| RF03794 | MIR5298 | MIPF0001286 |
| RF03795 | MIR6201 | MIPF0001960 |
| RF03796 | mir-5984 | MIPF0001468 |
| RF03797 | mir-9235 | MIPF0001906 |
| RF03798 | MIR4227 | MIPF0001115 |
| RF03799 | mir-3202 | MIPF0000846 |
| RF03800 | mir-4477 | MIPF0001274 |
| RF03801 | mir-7143 | MIPF0002024 |
| RF03802 | MIR5649 | MIPF0001369 |
| RF03803 | MIR1886 | MIPF0001647 |
| RF03804 | mir-4989 | MIPF0001239 |
| RF03805 | bpcv-mir-B1 | MIPF0001235 |
| RF03806 | mir-2007 | MIPF0002094 |
| RF03807 | mir-4890 | MIPF0001703 |
| RF03808 | mir-3957 | MIPF0001605 |
| RF03809 | mir-1203 | MIPF0000633 |
| RF03810 | mir-8932 | MIPF0002071 |
| RF03811 | mir-1896 | MIPF0001981 |
| RF03812 | mir-BART19 | MIPF0000983 |
| RF03813 | MIR8041 | MIPF0001773 |
| RF03814 | mir-2797 | MIPF0000911 |
| RF03815 | mir-3051 | MIPF0001114 |
| RF03816 | MIR6297 | MIPF0001754 |
| RF03817 | mir-3836 | MIPF0001231 |
| RF03818 | MIR7994 | MIPF0001763 |
| RF03819 | mir-Ro6-3 | MIPF0001628 |
| RF03820 | mir-5429 | MIPF0001315 |
| RF03821 | mir-2002 | MIPF0001989 |
| RF03822 | mir-3931 | MIPF0001585 |
| RF03823 | mir-1814 | MIPF0002102 |
| RF03824 | MIR5285 | MIPF0001292 |
| RF03825 | mir-2335 | MIPF0001971 |
| RF03826 | MIR825 | MIPF0001178 |
| RF03827 | mir-2229 | MIPF0001694 |
| RF03828 | MIR831 | MIPF0001132 |
| RF03829 | mir-1257 | MIPF0000998 |
| RF03830 | mir-1003 | MIPF0000923 |
| RF03831 | mir-627 | MIPF0000550 |
| RF03832 | mir-H4 | MIPF0001247 |
| RF03833 | mir-5928 | MIPF0001443 |
| RF03834 | mir-3173 | MIPF0001383 |
| RF03835 | mir-955 | MIPF0000989 |
| RF03836 | mir-3547 | MIPF0001788 |
| RF03837 | mir-3138 | MIPF0001428 |
| RF03838 | MIR8032 | MIPF0001653 |
| RF03839 | MIR2599 | MIPF0000913 |
| RF03840 | mir-3064 | MIPF0001238 |
| RF03841 | mir-8941 | MIPF0001932 |
| RF03842 | MIR2680 | MIPF0000841 |
| RF03843 | MIR2600 | MIPF0001284 |
| RF03844 | mir-3190 | MIPF0001251 |
| RF03845 | mir-3327 | MIPF0001547 |
| RF03846 | mir-4861 | MIPF0001822 |
| RF03847 | mir-1538 | MIPF0000903 |
| RF03848 | mir-1549 | MIPF0001345 |
| RF03849 | MIR1219 | MIPF0000346 |
| RF03850 | mir-961 | MIPF0001006 |
| RF03851 | mir-1002 | MIPF0000909 |
| RF03852 | mir-610 | MIPF0000661 |
| RF03853 | mir-2013 | MIPF0001777 |
| RF03854 | mir-1949 | MIPF0001139 |
| RF03855 | MIR5241 | MIPF0001290 |
| RF03856 | mir-1188 | MIPF0001188 |
| RF03857 | mir-3129 | MIPF0001458 |
| RF03858 | mir-1991 | MIPF0000732 |
| RF03859 | mir-6132 | MIPF0001438 |
| RF03860 | MIR1520 | MIPF0000581 |
| RF03861 | mir-4484 | MIPF0001440 |
| RF03862 | MIR5274 | MIPF0001311 |
| RF03863 | mir-1258 | MIPF0000684 |
| RF03864 | mir-BART9 | MIPF0000955 |
| RF03865 | mir-7637 | MIPF0001774 |
| RF03866 | mir-1889 | MIPF0000954 |
| RF03867 | mir-4081 | MIPF0001241 |
| RF03868 | mir-H2 | MIPF0001214 |
| RF03869 | mir-2834 | MIPF0001047 |
| RF03870 | mir-2040 | MIPF0000713 |
| RF03871 | mir-5681 | MIPF0001343 |
| RF03872 | MIR6426 | MIPF0001561 |
| RF03873 | mir-3192 | MIPF0001597 |
| RF03874 | mir-8834 | MIPF0002099 |
| RF03875 | MIR5272 | MIPF0001278 |
| RF03876 | mir-7132 | MIPF0002098 |
| RF03877 | mir-3937 | MIPF0001500 |
| RF03878 | mir-B6 | MIPF0001260 |
| RF03879 | mir-2831 | MIPF0000978 |
| RF03880 | mir-1684 | MIPF0001537 |
| RF03881 | mir-59 | MIPF0000940 |
| RF03882 | mir-4889 | MIPF0001686 |
| RF03883 | mir-9256 | MIPF0001895 |
| RF03884 | mir-725 | MIPF0001355 |
| RF03885 | mir-4178 | MIPF0000928 |
| RF03886 | mir-1993 | MIPF0000716 |
| RF03887 | mir-2511 | MIPF0000977 |
| RF03888 | mir-3160 | MIPF0001028 |
| RF03889 | mir-4776 | MIPF0001210 |
| RF03890 | mir-B8 | MIPF0002021 |
| RF03891 | mir-2545 | MIPF0000994 |
| RF03892 | mir-4860 | MIPF0001765 |
| RF03893 | mir-4879 | MIPF0001780 |
| RF03894 | mir-5736 | MIPF0001480 |
| RF03895 | MIR2679 | MIPF0000892 |
| RF03896 | MIR2275 | MIPF0000797 |
| RF03897 | mir-4077 | MIPF0000905 |
| RF03898 | mir-7616 | MIPF0001718 |
| RF03899 | mir-5912 | MIPF0001497 |
| RF03900 | mir-9242 | MIPF0002032 |
| RF03901 | mir-BART18 | MIPF0000948 |
| RF03902 | MIR7784 | MIPF0001728 |
| RF03903 | mir-679 | MIPF0001564 |
| RF03904 | MIR7991 | MIPF0001701 |
| RF03905 | MIR6149 | MIPF0001580 |
| RF03906 | mir-3199 | MIPF0001026 |
| RF03907 | mir-4428 | MIPF0001630 |
| RF03908 | MIR6459 | MIPF0001730 |
| RF03909 | mir-3804 | MIPF0001255 |
| RF03910 | mir-H5 | MIPF0000966 |
| RF03911 | mir-1910 | MIPF0000987 |
| RF03912 | mir-4659 | MIPF0001256 |
| RF03913 | mir-992 | MIPF0000914 |
| RF03914 | mir-BART21 | MIPF0000961 |
| RF03915 | MIR5286 | MIPF0001342 |
| RF03916 | MIR400 | MIPF0001157 |
| RF03917 | mir-4449 | MIPF0002103 |
| RF03918 | mir-4922 | MIPF0001229 |
| RF03919 | mir-1540 | MIPF0001772 |
| RF03920 | mir-237 | MIPF0000904 |
| RF03921 | MIR860 | MIPF0001176 |
| RF03922 | MIR2119 | MIPF0000762 |
| RF03923 | mir-964 | MIPF0000949 |
| RF03924 | mir-1228 | MIPF0000924 |
| RF03925 | mir-1011 | MIPF0000969 |
| RF03926 | MIR1435 | MIPF0000760 |
| RF03927 | MIR538 | MIPF0000161 |
| RF03929 | mir-6579 | MIPF0001492 |
| RF03930 | mir-1909 | MIPF0000973 |
| RF03931 | mir-2061 | MIPF0001790 |
| RF03932 | mir-1597 | MIPF0000764 |
| RF03933 | MIR6445 | MIPF0001621 |
| RF03934 | mir-m107-1 | MIPF0000446 |
| RF03935 | mir-763 | MIPF0000722 |
| RF03936 | mir-4079 | MIPF0001273 |
| RF03937 | mir-9456 | MIPF0001974 |
| RF03938 | mir-4662 | MIPF0001245 |
| RF03939 | mir-4868 | MIPF0001233 |
| RF03940 | MIR9563 | MIPF0001941 |
| RF03941 | mir-3158 | MIPF0001065 |
| RF03942 | mir-5727 | MIPF0001627 |
| RF03943 | mir-1824 | MIPF0001717 |
| RF03944 | mir-935 | MIPF0000606 |
| RF03945 | mir-4876 | MIPF0001776 |
| RF03946 | mir-3132 | MIPF0001509 |
| RF03947 | mir-658 | MIPF0000643 |
| RF03948 | mir-5045 | MIPF0001341 |
| RF03949 | mir-8830 | MIPF0001924 |
| RF03950 | mir-4928 | MIPF0001818 |
| RF03951 | MIR7725 | MIPF0001792 |
| RF03952 | mir-3718 | MIPF0001225 |
| RF03953 | mir-5350 | MIPF0001367 |
| RF03954 | mir-8993 | MIPF0002018 |
| RF03955 | MIR2934 | MIPF0001269 |
| RF03956 | mir-737 | MIPF0001689 |
| RF03957 | mir-5856 | MIPF0001503 |
| RF03958 | mir-959 | MIPF0000910 |
| RF03959 | mir-303 | MIPF0001013 |
| RF03960 | mir-6644 | MIPF0001504 |
| RF03961 | mir-9033 | MIPF0002087 |
| RF03962 | mir-958 | MIPF0000912 |
| RF03963 | mir-2996 | MIPF0001023 |
| RF03964 | mir-H1 | MIPF0000738 |
| RF03965 | mir-8927 | MIPF0001979 |
| RF03966 | mir-76 | MIPF0000902 |
| RF03967 | mir-1270 | MIPF0000877 |
| RF03968 | mir-8934 | MIPF0002010 |
| RF03969 | mir-622 | MIPF0000619 |
| RF03970 | mir-2766 | MIPF0001608 |
| RF03971 | mir-3572 | MIPF0001244 |
| RF03972 | mir-3150 | MIPF0001102 |
| RF03973 | mir-5615 | MIPF0001318 |
| RF03974 | MIR838 | MIPF0001184 |
| RF03975 | mir-5881 | MIPF0001420 |
| RF03976 | MIR898 | MIPF0000378 |
| RF03977 | mir-976 | MIPF0000916 |
| RF03978 | mir-1269 | MIPF0000984 |
| RF03979 | mir-2006 | MIPF0001936 |
| RF03980 | mir-2012 | MIPF0000720 |
| RF03981 | mir-8799 | MIPF0001881 |
| RF03982 | mir-4865 | MIPF0001750 |
| RF03983 | MIR5284 | MIPF0001277 |
| RF03984 | MIR2933 | MIPF0001072 |
| RF03985 | mir-9250 | MIPF0002073 |
| RF03986 | mir-6095 | MIPF0001433 |
| RF03987 | MIR6136 | MIPF0001741 |
| RF03988 | mir-6301 | MIPF0001556 |
| RF03989 | mir-7950 | MIPF0001678 |
| RF03990 | mir-4888 | MIPF0001724 |
| RF03991 | mir-9209 | MIPF0001927 |
| RF03992 | mir-6302 | MIPF0001398 |
| RF03993 | mir-8536 | MIPF0002030 |
| RF03994 | mir-7558 | MIPF0001716 |
| RF03995 | mir-7957 | MIPF0001668 |
| RF03996 | mir-3012 | MIPF0001018 |
| RF03997 | mir-8510 | MIPF0001886 |
| RF03998 | mir-2160 | MIPF0000769 |
| RF03999 | mir-7883 | MIPF0001849 |
| RF04000 | MIR8139 | MIPF0001665 |
| RF04001 | mir-7918 | MIPF0001655 |
| RF04002 | MIR6143 | MIPF0001685 |
| RF04003 | mir-4841 | MIPF0001264 |
| RF04004 | mir-7940 | MIPF0001719 |
| RF04005 | mir-4599 | MIPF0001252 |
| RF04006 | mir-9338 | MIPF0001921 |
| RF04007 | mir-8489 | MIPF0002034 |
| RF04008 | mir-4880 | MIPF0001833 |
| RF04009 | mir-754 | MIPF0000749 |
| RF04010 | mir-3003 | MIPF0000818 |
| RF04011 | mir-8515 | MIPF0002064 |
| RF04012 | MIR7532 | MIPF0001820 |
| RF04013 | mir-4818 | MIPF0001211 |
| RF04014 | mir-8338 | MIPF0002060 |
| RF04015 | MIR946 | MIPF0000427 |
| RF04016 | mir-4873 | MIPF0001806 |
| RF04017 | mir-4864 | MIPF0001702 |
| RF04018 | mir-5316 | MIPF0001364 |
| RF04019 | MIR950 | MIPF0000407 |
| RF04020 | mir-4859 | MIPF0001784 |
| RF04021 | mir-3492 | MIPF0001622 |
| RF04022 | mir-4863 | MIPF0001778 |
| RF04023 | MIR1314 | MIPF0001609 |
| RF04024 | mir-9571 | MIPF0002068 |
| RF04025 | mir-7964 | MIPF0001749 |
| RF04026 | mir-8499 | MIPF0002029 |
| RF04027 | mir-1422 | MIPF0000565 |
| RF04028 | mir-7948 | MIPF0001695 |
| RF04029 | mir-8521 | MIPF0001871 |
| RF04030 | mir-6096 | MIPF0001436 |
| RF04031 | mir-2686 | MIPF0000927 |
| RF04032 | MIR2912 | MIPF0000778 |
| RF04033 | mir-8512 | MIPF0002076 |
| RF04035 | mir-7910 | MIPF0001840 |
| RF04036 | mir-2076 | MIPF0001795 |
| RF04037 | mir-7911 | MIPF0001858 |
| RF04038 | mir-5985 | MIPF0001491 |
| RF04039 | mir-2242 | MIPF0000945 |
| RF04040 | mir-4866 | MIPF0001756 |
| RF04041 | MIR6154 | MIPF0001560 |
| RF04042 | mir-7961 | MIPF0001832 |
| RF04043 | mir-2062 | MIPF0001753 |
| RF04044 | mir-7939 | MIPF0001646 |
| RF04045 | mir-2147 | MIPF0000751 |
| RF04046 | mir-2154 | MIPF0000781 |
| RF04047 | mir-9228 | MIPF0001876 |
| RF04048 | mir-7927 | MIPF0001660 |
| RF04049 | MIR7127 | MIPF0001408 |
| RF04050 | mir-2072 | MIPF0001697 |
| RF04051 | mir-8517 | MIPF0002077 |
| RF04052 | mir-466 | MIPF0000208 |
| RF04053 | mir-95 | MIPF0000098 |
| RF04054 | mir-2944 | MIPF0001335 |
| RF04055 | mir-1261 | MIPF0000802 |
| RF04056 | mir-982 | MIPF0000843 |
| RF04057 | MIR1432 | MIPF0001063 |
| RF04059 | MIR5020 | MIPF0001226 |
| RF04060 | mir-703 | MIPF0000761 |
| RF04061 | mir-1420 | MIPF0000574 |
| RF04062 | MIR949 | MIPF0001455 |
| RF04063 | MIR2629 | MIPF0000824 |
| RF04064 | MIR5267 | MIPF0001276 |
| RF04065 | mir-7563 | MIPF0001709 |
| RF04066 | mir-1256 | MIPF0000594 |
| RF04067 | mir-4520 | MIPF0001272 |
| RF04068 | mir-3927 | MIPF0001462 |
| RF04069 | MIR6135 | MIPF0001644 |
| RF04070 | MIR6440 | MIPF0001406 |
| RF04071 | MIR2863 | MIPF0000925 |
| RF04072 | mir-7386 | MIPF0001658 |
| RF04073 | mir-727 | MIPF0001371 |
| RF04074 | mir-1199 | MIPF0001659 |
| RF04075 | mir-1545 | MIPF0001711 |
| RF04076 | mir-H11 | MIPF0000833 |
| RF04077 | mir-1245 | MIPF0000620 |
| RF04078 | mir-1905 | MIPF0000753 |
| RF04079 | MIR161 | MIPF0000455 |
| RF04080 | mir-762 | MIPF0000624 |
| RF04081 | mir-1992 | MIPF0000705 |
| RF04082 | mir-54 | MIPF0000874 |
| RF04083 | MIR528 | MIPF0000868 |
| RF04084 | mir-595 | MIPF0000651 |
| RF04085 | mir-1271 | MIPF0000483 |
| RF04087 | MIR5565 | MIPF0001279 |
| RF04088 | MIR812 | MIPF0000345 |
| RF04089 | mir-753 | MIPF0000752 |
| RF04090 | mir-6529 | MIPF0001771 |
| RF04091 | mir-72 | MIPF0000277 |
| RF04092 | MIR1508 | MIPF0000719 |
| RF04093 | mir-1784 | MIPF0001873 |
| RF04094 | MIR537 | MIPF0000207 |
| RF04095 | MIR9746 | MIPF0001884 |
| RF04096 | mir-4433 | MIPF0001826 |
| RF04097 | MIR2630 | MIPF0000814 |
| RF04098 | MIR9471 | MIPF0002045 |
| RF04099 | MIR837 | MIPF0001155 |
| RF04100 | mir-493 | MIPF0000230 |
| RF04101 | mir-8864 | MIPF0001919 |
| RF04102 | MIR6457 | MIPF0001534 |
| RF04103 | mir-8356 | MIPF0001997 |
| RF04104 | MIR918 | MIPF0000374 |
| RF04105 | mir-3165 | MIPF0001635 |
| RF04106 | mir-1303 | MIPF0000608 |
| RF04107 | mir-5391 | MIPF0001944 |
| RF04108 | mir-2513 | MIPF0000980 |
| RF04109 | mir-1305 | MIPF0000965 |
| RF04110 | MIR5084 | MIPF0002026 |
| RF04111 | MIR6274 | MIPF0001706 |
| RF04112 | MIR5512 | MIPF0001478 |
| RF04113 | MIR1523 | MIPF0001327 |
| RF04114 | MIR4374 | MIPF0001152 |
| RF04115 | mir-6130 | MIPF0001413 |
| RF04116 | MIR9672 | MIPF0001942 |
| RF04117 | MIR5200 | MIPF0001904 |
| RF04118 | MIR9481 | MIPF0002096 |
| RF04119 | MIR1858 | MIPF0000650 |
| RF04120 | MIR5998 | MIPF0001613 |
| RF04121 | mir-1289 | MIPF0000626 |
| RF04122 | mir-1204 | MIPF0000671 |
| RF04123 | mir-3127 | MIPF0001439 |
| RF04124 | MIR8565 | MIPF0001889 |
| RF04125 | MIR9408 | MIPF0002027 |
| RF04126 | mir-9198 | MIPF0001887 |
| RF04127 | MIR8007 | MIPF0001839 |
| RF04128 | MIR8562 | MIPF0001861 |
| RF04129 | MIR4240 | MIPF0001198 |
| RF04130 | MIR7996 | MIPF0001691 |
| RF04131 | mir-2032 | MIPF0000736 |
| RF04132 | mir-4436 | MIPF0001236 |
| RF04133 | MIR2646 | MIPF0001010 |
| RF04134 | MIR7504 | MIPF0001798 |
| RF04135 | MIR5062 | MIPF0001922 |
| RF04136 | MIR1862 | MIPF0000580 |
| RF04137 | MIR834 | MIPF0001183 |
| RF04138 | mir-9243 | MIPF0001926 |
| RF04139 | MIR841 | MIPF0001112 |
| RF04140 | mir-3118 | MIPF0001928 |
| RF04141 | MIR8622 | MIPF0001931 |
| RF04142 | mir-1299 | MIPF0000625 |
| RF04143 | MIR869 | MIPF0001167 |
| RF04144 | MIR9783 | MIPF0001947 |
| RF04145 | MIR8706 | MIPF0001953 |
| RF04146 | mir-4742 | MIPF0001552 |
| RF04147 | MIR6224 | MIPF0001435 |
| RF04148 | MIR844 | MIPF0001197 |
| RF04149 | MIR447 | MIPF0000170 |
| RF04150 | MIR2655 | MIPF0000813 |
| RF04151 | MIR4387 | MIPF0001109 |
| RF04152 | mir-3618 | MIPF0001710 |
| RF04153 | MIR856 | MIPF0001162 |
| RF04154 | mir-1277 | MIPF0001937 |
| RF04155 | mir-9236 | MIPF0001905 |
| RF04156 | MIR2670 | MIPF0000855 |
| RF04157 | MIR9486 | MIPF0002054 |
| RF04158 | mir-568 | MIPF0000408 |
| RF04159 | MIR1437 | MIPF0001762 |
| RF04160 | MIR868 | MIPF0001173 |
| RF04161 | mir-3688 | MIPF0001263 |
| RF04162 | MIR9662 | MIPF0002119 |
| RF04163 | mir-8364 | MIPF0001877 |
| RF04164 | MIR1507 | MIPF0000699 |
| RF04165 | mir-2483 | MIPF0001532 |
| RF04166 | mir-2278 | MIPF0001522 |
| RF04167 | mir-1972 | MIPF0001025 |
| RF04168 | MIR839 | MIPF0001143 |
| RF04169 | mir-2235 | MIPF0000825 |
| RF04170 | MIR7723 | MIPF0001739 |
| RF04171 | MIR1509 | MIPF0000771 |
| RF04172 | MIR8675 | MIPF0002106 |
| RF04173 | MIR847 | MIPF0001171 |
| RF04174 | MIR4208 | MIPF0001108 |
| RF04175 | mir-1260b | MIPF0001381 |
| RF04176 | mir-3149 | MIPF0001935 |
| RF04185 | MIR2118_2 | MIPF0001409 |
| RF04186 | mir-278_2 | MIPF0000728 |
| RF04187 | MIR862 | MIPF0001145 |
| RF04193 | mir-51 | MIPF0000268 |
| RF04194 | mir-57 | MIPF0000271 |
| RF04195 | MIR6217 | MIPF0001550 |
| RF04196 | MIR5638 | MIPF0001350 |
| RF04197 | mir-9457 | MIPF0002122 |
| RF04198 | MIR8670 | MIPF0002041 |
| RF04199 | MIR6027 | MIPF0001424 |
| RF04200 | mir-9437 | MIPF0001943 |
| RF04201 | MIR7510 | MIPF0001684 |
| RF04202 | MIR5380 | MIPF0001331 |
| RF04203 | MIR1144 | MIPF0000432 |
| RF04204 | mir-3473 | MIPF0001230 |
| RF04205 | MIR2606 | MIPF0000888 |
| RF04206 | MIR9555 | MIPF0002081 |
| RF04207 | MIR5048 | MIPF0001453 |
| RF04208 | MIR8742 | MIPF0002016 |
| RF04209 | MIR7533 | MIPF0001834 |
| RF04210 | MIR8643 | MIPF0002008 |
| RF04211 | MIR5185 | MIPF0001246 |
| RF04212 | mir-8904 | MIPF0001977 |
| RF04213 | MIR7807 | MIPF0002091 |
| RF04214 | MIR7526 | MIPF0001651 |
| RF04215 | mir-9214 | MIPF0002108 |
| RF04216 | mir-509 | MIPF0000130 |
| RF04217 | mir-297 | MIPF0000204 |
| RF04223 | MIR2619 | MIPF0001294 |
| RF04224 | mir-9229 | MIPF0001875 |
| RF04225 | MIR7502 | MIPF0001869 |
| RF04226 | mir-9186 | MIPF0001868 |
| RF04227 | mir-9215 | MIPF0001893 |
| RF04228 | MIR6140 | MIPF0001666 |
| RF04229 | mir-9279 | MIPF0001911 |
| RF04230 | mir-9261 | MIPF0001938 |
| RF04231 | mir-9191 | MIPF0001987 |
| RF04232 | mir-9318 | MIPF0002056 |
| RF04233 | mir-680 | MIPF0000338 |
| RF04234 | mir-1421 | MIPF0000564 |
| RF04235 | mir-242_2 | MIPF0000731 |
| RF04236 | mir-1285 | MIPF0000559 |
| RF04237 | mir-1490 | MIPF0000525 |
| RF04238 | mir-4012 | MIPF0000964 |
| RF04239 | mir-4679 | MIPF0001228 |
| RF04240 | mir-4716 | MIPF0001476 |
| RF04241 | mir-5014 | MIPF0001634 |
| RF04242 | mir-5595 | MIPF0001590 |
| RF04243 | MIR169_3 | MIPF0000253 |
| RF04244 | MIR169_6 | MIPF0001058 |
| RF04245 | MIR169_7 | MIPF0001690 |
| RF04246 | MIR169_8 | MIPF0001712 |
| RF04248 | MIR7486 | MIPF0001682 |
| RF04249 | mir-310 | MIPF0000566 |
| RF04250 | mir-236 | MIPF0000232 |
| RF04251 | MIR5070 | MIPF0002013 |
| RF04252 | mir-8186 | MIPF0002035 |
| RF04254 | mir-1677 | MIPF0000849 |
| RF04255 | MIR8001 | MIPF0001852 |
| RF04257 | mir-9230 | MIPF0002035 |
| RF04258 | mir-994 | MIPF0001045 |
| RF04259 | mir-2003 | MIPF0002049 |
| RF04260 | mir-2005 | MIPF0001956 |
| RF04261 | MIR1861 | MIPF0000567 |
| RF04262 | MIR1319 | MIPF0001448 |
| RF04263 | MIR2593 | MIPF0000733 |
| RF04264 | mir-5408 | MIPF0001313 |
| RF04265 | mir-4650 | MIPF00001234 |
| RF04266 | mir-9412 | MIPF0001891 |
| RF04267 | mir-362 | MIPF0000209 |
| RF04268 | mir-512 | MIPF0000518 |
| RF04269 | mir-373 | MIPF0000500 |
| RF04270 | mir-743 | MIPF0000386 |
| RF04271 | mir-1244 | MIPF0000569 |
| RF04272 | mir-8908 | MIPF0001890 |
| RF04273 | MIR827_2 | MIPF0001769 |
| RF04274 | mir-9223 | MIPF0001885 |
| RF04275 | mir-2733 | MIPF0000766 |
| RF04276 | MIR4371 | MIPF0001141 |
| RF04277 | mir-1268 | MIPF0000946 |
| RF04278 | MIR4372 | MIPF0001257 |
| RF04279 | mir-H20 | MIPF0001760 |
| RF04280 | mir-890 | MIPF0000386 |
| RF04281 | mir-8791 | MIPF0001882 |
| RF04282 | mir-507 | MIPF0000130 |
| RF04283 | mir-3135 | MIPF0001219 |
| RF04284 | mir-532 | MIPF0000113 |
| RF04285 | mir-660 | MIPF0000113 |
| RF04286 | mir-513 | MIPF0000130 |
| RF04287 | mir-9195 | MIPF0001939 |
| RF04288 | mir-9201 | MIPF0001898 |
| RF04289 | mir-3596 | MIPF0001194 |
| RF04290 | mir-1197 | MIPF0000126 |
| RF04291 | mir-368 | MIPF0000091 |
| RF04292 | mir-379 | MIPF0000126 |
| RF04293 | mir-889 | MIPF0000514 |
| RF04294 | mir-3578 | MIPF0001166 |
| RF04295 | mir-329 | MIPF0000110 |
| RF04296 | mir-485 | MIPF0000201 |
| RF04297 | mir-35_2 | MIPF0001648 |
| RF04298 | mir-36_2 | MIPF0001664 |
| RF04299 | MIR814 | MIPF0000351 |
| RF04300 | mir-39 | MIPF0000304 |
| RF04301 | mir-200 | MIPF0000491 |
| RF04302 | mir-506 | MIPF0000130 |
| RF04303 | MIR162_2 | MIPF0000169 |

Table S1: The mapping between Rfam accessions, ids and miRBase family accessions.
